# From Character Strengths to Children’s Well-Being: Development and Validation of the Character Strengths Inventory for Elementary School Children

**DOI:** 10.3389/fpsyg.2018.02123

**Published:** 2018-11-02

**Authors:** Anat Shoshani, Lior Shwartz

**Affiliations:** Baruch Ivcher School of Psychology, Interdisciplinary Center Herzliya, Herzliya, Israel

**Keywords:** character strengths, children, well-being, mental-health, school, inventory

## Abstract

Although research on character strengths has flourished in recent years, the paucity of suitable quantitative instruments for the assessment of children’s character strengths limits the study of character development in childhood. The Character Strengths Inventory for Children (CSI-C) is a new self-report character inventory for children that was designed for easy administration directly to elementary school-aged children. The CSI-C provides an evaluation of 24 character strengths defined in Peterson and Seligman’s Values in Action Classification of Strengths. Data from two samples of 2,061 Israeli children aged 7–12 support the constructs of the instrument. Principal component analysis and confirmatory factor analysis of the 96 CSI-C items revealed preliminary evidence for a hierarchical structure with 24 lower factors nested within four higher-order latent factors: interpersonal, transcendence, intellectual, and temperance strengths. Children’s interpersonal and temperance strengths were negatively associated with mental health difficulties, and their temperance and transcendence strengths were positively associated with subjective well-being. The intellectual and temperance strengths were correlated with children’s school functioning and grit. The potential uses of the CSI-C in research and practice are discussed.

## Introduction

Character education has been a central feature of children’s socialization from the dawn of history to the present day. Most parents want to instill foundational values and virtues in their children. They aim to raise children who have desirable, praiseworthy personality traits and whose characters are imbued with a strong ethical compass (Delattre and Russell, 1993).

Despite the widespread belief that character is a pillar of positive youth development, there is little research and few instruments to measure individual differences in character among children in elementary school, and little agreement about how character should be conceptualized during this period of development (Berkowitz and Bier, 2004). One of the major obstacles to conducting research on children’s character is the difficulty of devising reliable ways to measure individual differences that correspond to the reading level, concrete thinking, and partial self-awareness of children. As a result, vital questions about character formation and how it relates to the flourishing of young children remain unanswered.

Peterson and Seligman’s (2004) watershed publication introduced the Values in Action (VIA) classification that defined good character in terms of 24 character strengths and six universal virtues. The six are humanity, wisdom and knowledge, justice, courage, transcendence and temperance, which were identified from an extensive range of literatures around the world by philosophers and religious leaders (Peterson and Seligman, 2004). The 24 character strengths were conceptualized as the mechanisms and processes that exemplify or lead to these six virtues. Since their publication, a number of instruments have been developed to assess character strengths in late childhood and adulthood. However, there are no inventories specifically designed for elementary school children.

This study addresses this gap by empirically validating an inventory based on the VIA classification of strengths to measure individual differences in character in elementary school-aged children (aged 7–12). It also examined the relationships between the manifestations of character strengths in middle childhood and social, emotional, and academic outcomes.

### The Conceptualization of Character

Character is a complex construct, and has been defined in a multitude of ways to capture the breadth of its totality. It is commonly described as a unified set of good personality traits or a collection of individual virtues (Elliott, 2004). It is typified by an enduring positive dispositional constellation of habits such as moral commitment, social responsibility, resoluteness and self-discipline against which the individual as a whole is judged to be adequate, deficient, or exemplary (Baumrind, 1989). Hay et al. (1995) defined character as individuals’ general attitude toward their societal responsibilities and receptivity to their surroundings, which is supported by knowledge of social conventions, emotional reactions to others’ distress, and the development of prosocial skills (Hay et al., 1995, p. 24). Character has also been seen as involving engagement in morally relevant stances or conduct (Wynne and Walberg, 1984).

Though researchers have primarily seen character as the moral quality of a person, others consider that character also comprises certain attributes and traits that are not necessarily moral in nature but nevertheless have moral functions. Berkowitz (1997) distinguished between moral and non-moral qualities of character. “Non-moral” traits such as creativity, courage and perseverance support moral action and can serve to enact moral behavior for instance by mustering courage to defend the rights of minority groups in situations of discrimination. In line with this perspective, Peterson and Seligman (2004) also posited that the scope of character exceeds simply doing what is moral, but involves engaging in virtuous behavior in pursuit of the important things in life. In their classification of character strengths, some fall within the “intellectual” category such as creativity, curiosity, open-mindedness, love of learning, and perspective. These character strengths reflect the virtue of wisdom and the exceptional capacity to use knowledge and judgment in the “service of the good life” (Peterson and Seligman, 2004, p. 96).

Thus overall, conceptualizations of character development in childhood appear to rely on cultivating fundamental social emotional and cognitive skills. This includes the ability to recognize others’ perspectives, engage in prosocial behavior, the ability to distinguish between right and wrong, the acquisition of societal standards, and the ability to possess and use knowledge that contributes to a sense of well-being and a meaningful life.

### The VIA Classification of Strengths and Virtues

Just as there is no consensus on the definition of character, there are also different perspectives on the components or the best traits to be developed, nurtured, and maintained to shape children’s overall good character. Although there has been a recent resurgence of character education programs in schools and numerous after-school youth development projects, only a few studies have addressed what makes up good character in middle childhood (Berkowitz and Bier, 2004), and even fewer have investigated its potential impact on young children’s mental health and functioning (Shoshani and Slone, 2016).

In one of the rare studies that did examine the components of children’s character, Lamb (1993) suggested the three key characteristics were empathy, awareness of standards, and prosocial behavior (altruism). Several years later, Berkowitz and Grych (1998) identified eight core aspects of children’s character which they defined as empathy, social orientation, conscience, compliance, moral reasoning, self-esteem, honesty and altruism. Later, the VIA Institute on Character (Dahlsgaard et al., 2005) expanded this list based on the results of a large-scale comparative study, and put forward what is considered the most comprehensive and systematic model to date of the core components of human character.

The VIA Institute perspective posits that character is made up of varying, yet relatively stable personality traits that are malleable and develop differently depending on the specific social and cultural realities in which the child is raised and educated (Peterson and Seligman, 2004). The VIA conceptualization provides a hierarchical classification of two main positive aspects of good character that reflect two conceptual levels: character strengths and virtues. Virtues are described as acquired qualities that enable people to flourish or to live a good life (Mintz, 1996).

Character strengths are conceptualized as the psychological traits that enable the individual to display virtues (Park, 2004). For example, by exhibiting strengths such as gratitude and hope, people can express the virtue of transcendence. The VIA identified a list of 24 character strengths that can be expressed in human emotion, behavior, and thought (Park and Peterson, 2006b). This final set of strengths was the result of a comprehensive process that involved dozens of scholars and psychologists, over the course of several years which implemented brainstorming and systematic reviews of the contemporary and historical literature on strengths and virtues (McGrath, 2014; McGrath and Walker, 2016).

This hierarchical structure led to the final VIA classification which is comprised of the set of the 24 character strengths, organized under the six broad categories of virtues. Humanity includes strengths that reflect collectivism, convivial relations with others and communion (e.g., kindness, social intelligence, love). The virtue of Justice consists of strengths that favor optimal and synergistic interactions between individuals and their groups (e.g., fairness, teamwork, leadership). Temperance is comprised of strengths that reflect the modulation of behavior, emotion, and motivation (e.g., modesty, self-regulation, forgiveness, prudence). Wisdom taps strengths that are related to appreciating and seeking out knowledge and using it with good judgment (e.g., curiosity, creativity, love of learning). Transcendence is related to valuing and pursuing a purpose, higher meaning, or connection (e.g., hope, gratitude, humor, spirituality). Courage is composed of strengths that entail the exercise of desire to accomplish goals and to engage in continuous improvement in the face of challenges and difficulties (e.g., persistence, zest, bravery).

The VIA character strengths are thought to be universal positive traits in different societies and explain people’s wellbeing (Peterson and Seligman, 2004). It assumes these strengths are pervasive in human nature, and are reflected in common values, virtues and dispositions people need to survive and flourish, and are therefore held and manifested by most individuals in most societies (Bok, 1995; Peterson and Seligman, 2004). An alternative view claims that cultural worldviews and values related to happiness and the nature of the self may give more weight to certain strengths than others as a function of culture and contexts (Wong, 2013). For example, drawing on the Big Five classification (Hofstede, 2001), Western societies tend to emphasize individualism, autonomy, and personal goals whereas Eastern societies tend to emphasize collectivism community cooperation, and group cohesion (Matsumoto and Kupperbusch, 2001). These presumed differences may influence which character strengths are valued and come to the fore in different societies (Wong, 2013).

There is empirical evidence for both the universal and the cultural-specific theories of character strengths and the contribution of specific strengths to subjective well-being. For example, Park et al. (2006) found significant similarities in the relative endorsement of the 24 VIA strengths in adults from 54 different nations. The US character strengths profile converged with those of 53 other nations and across the 50 US states in the sample, with minor differences in religiousness. The character strengths that were the most commonly endorsed as signature strengths (i.e., participants’ five top- ranked strengths) were fairness, kindness, gratitude, honesty, and open-mindedness. Modesty, prudence, and self-regulation were rated the lowest overall in the countries surveyed (Park et al., 2006).

To extend generalizations to three very different cultures, Biswas-Diener (2006) explored VIA character strengths in Maasai, Northern Greenland Inughuit, and Native American participants. The participants were asked to indicate whether each strength existed as a concept in their society, rate the importance of the strength to society, and state whether they would like their children to have that specific strength. Findings showed high rates of agreement across these cultures on the desirability, existence, and development of the 24 character strengths. Despite these strong similarities, there were differences in the perceived importance of certain strengths (such as perspective, modesty, love of learning), and the existence of social institutions that facilitate each strength. Among the Maasai, several strengths, such as fairness, modesty, and open mindedness were thought to apply to children less often. Many Inughuit felt that children do not exhibit a large number of these strengths.

### The VIA Classification Measurement

Two inventories have been developed to date on the basis of the VIA theoretical classification, and are designed to be used on adults (the VIA-IS; Peterson et al., 2005) and adolescents (the VIA-Youth; Park and Peterson, 2006b). Factor analysis of the inventories has yielded a three to five factor structure across several studies which conflicts with the theoretical six-factor model (McGrath and Walker, 2016). In fact, inconsistent findings including five-factor models (Peterson and Seligman, 2004; Ruch et al., 2010; Singh and Choubisa, 2010; Littman-Ovadia and Lavy, 2011), four-factor models (Macdonald et al., 2008; Brdar and Kashdan, 2010), and three-factor models (Khumalo et al., 2008; Shryack et al., 2010; Duan et al., 2012, 2013; Duan and Bu, 2017) have been obtained from samples of participants from different cultures, such as United States, India, China, Germany, Australia, Africa, Israel, and Croatia.

For example, in studies on American adolescents, Park and Peterson (2006b) identified four factors in the VIA-Youth, which they termed Temperance, Intellectual, Other-Directed, and Transcendence. Shoshani and Slone (2013) and Shoshani (2018) found similar factors in children and adolescent samples in Israel. Other studies reported a fifth factor of Vitality (Toner et al., 2012) or Leadership (Gillham et al., 2011; Ruch et al., 2014) in Australian, German, and American samples. In interpreting these inconsistencies, it has been suggested that the same character strength might serve a different function in different cultural environments (e.g., Duan et al., 2012; Wong, 2013).

The VIA-Youth has been applied to samples of children over age 10 and to adolescents. Less attention has been paid to the age-specific manifestations of the 24 character strengths in younger school children, and to the appropriateness of these items to cognitive abilities and reading level at the beginning of elementary school. In one study that did directly address the character features of a sample of United States children aged 3–9, parents were asked to write free descriptions of their children’s character strengths (Park and Peterson, 2006a). Their verbal descriptions were coded qualitatively and quantitatively on the basis of the VIA classification. It was clear from these descriptions that some character strengths manifest differently in young children than in adolescents and adults, both qualitatively and quantitatively. For example, according to the parents’ descriptions, all 24 character strengths existed in their children’s personalities, although some of them appeared more frequently, including love, kindness, zest, humor, curiosity, and creativity. In contrast, character strengths which are presumed to depend on cognitive or emotional maturation such as forgiveness, open-mindedness, authenticity, modesty, and gratitude were not frequently mentioned.

In many cases, the parents only provided simple behavioral expressions of specific strengths as opposed to more complex depictions of manifestations at later ages. For example, they described their children’s kindness in terms of concrete cooperating or helping behaviors such as “helping out around the house” rather than more general altruistic tendencies, capacities for caring or generosity. These observations may reflect the principle of “cumulative continuity” in personality trait development (Roberts and DelVecchio, 2000) which refers to sequential changes in cognitive, behavioral, interpersonal, and emotional facets of a specific personality trait that transition from more basic to more sophisticated and stable qualities from infancy through childhood to adulthood (Roberts and DelVecchio, 2000; Soto and Tackett, 2015).

Studies on character strengths in Israel, the specific context of this study, have validated the Hebrew version of the VIA-IS (Littman-Ovadia and Lavy, 2011) and VIA-Youth (Shoshani and Slone, 2013). Littman-Ovadia and Lavy (2011) found that the means, standard deviations, scale reliabilities, and associations with life satisfaction on the VIA-IS in the Israeli sample were consistent with findings in the United States and United Kingdom (Park et al., 2004; Linley et al., 2007). A study by Shoshani and Slone (2013) on the contribution of character strengths to Israeli adolescents’ school adjustment and subjective well-being during the pivotal period of middle school transition also found high similarities to findings on American and German adolescents (Park and Peterson, 2006b; Gillham et al., 2011; Ruch et al., 2014). Shoshani (2018) also recently developed a parent report inventory for the assessment of the VIA character strengths in preschool children, and validated it in two large samples of 3- to 6-year olds in Israel. Parental reports provided evidence for the expressions of all 24 character strengths in early childhood and their associations with children’s emotional well-being. These findings support the use of the VIA classification system in Israel, and also support the claims made by Park et al. (2006) that the VIA classification of strengths reflects a universal aspect of human nature.

### The Present Study

Using the VIA classification of strengths, the present study was designed to assess The Character Strengths Inventory for Children; CSI-C, a character strengths self-report inventory suitable for children aged 7–12. Its associated goal was to then examine the associations between children’s character strengths and their mental health as reflected in self-reports of their socio-emotional difficulties and pro-sociality, as well as their subjective well-being, as measured by their expressed degree of life satisfaction and positive or negative emotions. The contribution of children’s strengths to their school engagement was assessed on a cognitive engagement measure that evaluated their psychological investment in learning tasks. A grit scale explored children’s ability to pursue long-term goals with sustained effort and interest over time (Duckworth et al., 2007).

In line with previous findings, we expected that intellectual, temperance and interpersonal strengths would be strong predictors of children’s mental health, subjective wellbeing, and school engagement (Gillham et al., 2011; Shoshani and Slone, 2013; Shoshani and Slone, 2017). Studies have reported moderate to strong correlations between most of the VIA character strengths and components related to subjective well-being such as purpose, self-acceptance, mastery, and mental health (Leontopoulou and Triliva, 2012), school success (Shoshani and Aviv, 2012; Shoshani and Slone, 2013; Wagner and Ruch, 2015), and fewer mental health symptoms, greater life satisfaction, (Park, 2004; Gillham et al., 2011; Shoshani and Aviv, 2012; Wagner and Ruch, 2015), and better resilience (Shoshani and Slone, 2016) in adolescents.

## Materials and Methods

### Participants

A sample of 2,061 participants was recruited from elementary schools in several geographically and economically diverse cities in the center of Israel, with the assistance of the municipal education departments. Twenty-two participants that did not fill out all the scales, and six participants that had missing data on the CSI-C were excluded from the analyses. Therefore, the final sample consisted of 2,033 children. This sample was randomly divided into two sub-samples for exploratory and confirmatory factor analyses. Sample 1 was composed of 1,010 children, aged 7–12 (524 boys and 486 girls; mean age = 9.93, *SD* = 1.59) from four elementary schools. The children were mostly Jewish (97%) and Israeli born (98%). The majority of the children’s parents were married (84%), 13% were divorced or separated, 2% were single, and 1% were widowed. In addition, 58% of the children reported middle socioeconomic status, 24% high SES, and 18% low SES. Sample 2 was composed of 1,023 school children aged 7–12 (mean age = 9.48, *SD* = 1.69; 506 boys and 517 girls) from another four elementary schools. These children were predominantly Israeli-born (99%), most of whom were Jewish (98%), and were relatively homogeneous in terms of SES, with mostly (61%) middle SES, 20% high SES, and 19% low SES. At the time of assessment, 83% of the children’s parents were married, 14% were divorced or separated, 2% were single, and 1% were widowed.

### Procedure

After receiving academic and municipal ethics committee authorizations and the school principals’ consent, parents were e-mailed active parental consent forms for their children’s participation and the socio-demographic questionnaire. The children themselves provided their oral consent. All parents except two gave written consent for participation of their children in the study. Research assistants administered the battery of questionnaires to the school children in their classrooms using tablets and the Qualtrics Offline Surveys application, thus allowing us to counterbalance the order of the questionnaires across subjects.

### Measures

#### The Character Strengths Inventory for Children; CSI-C

The development of the CSI-C was both theory-driven (i.e., based on the scientific literature on character strengths and child development), and empirically driven (i.e., based on interviews with children, parents, and teachers about the children’s daily behaviors that reflect the 24 character strengths). The inventory development team was composed of an experienced character strengths researcher, Ph.D. and MA students, and clinical child psychologists. All took part in an academic seminar on the VIA classification of strengths devoted to this research project.

The CSI-C was developed by writing developmentally and age appropriate items that covered the 24 character strengths in the VIA Classification. The items are written in simple language, without metaphors or idioms, and refer to situations and settings familiar to children; e.g., family, school, and friends. The inventory items were refined over a 2-year period on the basis of commentary from clinical and developmental psychologists, teachers, and feedback from focus groups of parents and children. In developing the list of potential items for the CSI-C, the team avoided writing items that were too long or complex for children’s comprehension and memory, or using words that were beyond their vocabulary level. An initial item pool of 120 items was chosen, to keep the questionnaire reasonably short for children, and to maximize response rate. The CSI-C was developed in Hebrew and in English to facilitate cross-cultural validation of the new instrument, and was validated for the Hebrew version in this study.

The 120 items representing the 24 character strengths of the VIA classification of strengths were tested on a sample of 176 elementary school children aged 7–12.

Each strength scale was analyzed and 18 items that were complex or ambiguous, loaded strongly onto more than one factor, or had a factor loading below 0.40, were eliminated and replaced with new items. In addition, several items were rewritten to clarify meaning and to distinguish them from other items that had a similar meaning.

The final version that was used in this study consisted of 120 items. Each one of the 24 strengths scales is measured by five items. Items are rated on a five-point Likert scale (1 = not like me at all; 2 = a little like me; 3 = somewhat like me; 4 = mostly like me; 5 = very much like me). Information on psychometric properties of the inventory is presented in the “Results” section.

#### Strengths and Difficulties Questionnaire (SDQ; Goodman et al., 1998)

The Strengths and Difficulties Questionnaire (SDQ) is a widely used self-report questionnaire on child mental health that assesses behavioral and emotional problems in children aged 3–16. The questionnaire comprises 25 items forming five subscales: Emotional symptoms, Conduct problems, Hyperactivity, Peer problems, and Prosocial behavior. Items are scored on a 3-point Likert scale ranging from 1 (not true) to 3 (certainly true). The Hebrew version of the inventory used in this study has demonstrated excellent criterion validity for children (Mansbach-Kleinfeld et al., 2010; Shoshani et al., 2016a; Shoshani and Russo-Netzer, 2017). The Cronbach’s alpha coefficients for the subscales in this study ranged from 0.73 to 0.87.

#### Satisfaction With Life Scale Adapted for Children (SWLS-C; Gadermann et al., 2010)

This five-item scale was adapted for children from the SWLS (Diener et al., 1985), which is one of the most commonly used scales to assess life satisfaction in adults. The scale assesses the child’s satisfaction with his or her life as a whole using a seven-point Likert scale, ranging from strongly disagree (1) to strongly agree (7). A previous study provides evidence for good reliability and validity of the Hebrew version of the scale (Shoshani and Russo-Netzer, 2017). In this study, the SWLS-C yielded a satisfactory Cronbach’s alpha (alpha coefficient = 0.77).

#### The Positive and Negative Affect Schedule for Children (PANAS-C; Ebesutani et al., 2012)

This scale was developed to examine levels of positive and negative emotions the child has experienced over the previous few weeks. The PANAS-C-P consists of 10 adjectives that describe five positive emotions (e.g., excited, enthusiastic, proud) and five negative emotions (upset, afraid, nervous). Responses are expressed on a scale ranging from 1 (very slightly or not at all) to 5 (extremely). The Hebrew version of the scale has shown good internal consistency (Shoshani et al., 2016a; Shoshani and Kanat-Maymon, 2018). The Cronbach’s alphas in this study were 0.80 and 0.76 for the positive and negative affect subscales, respectively.

#### Cognitive School Engagement (The National Center for School Engagement [NCSE], 2006)

Children’s school cognitive engagement was measured by the Hebrew version of a 22 item scale that measures the efforts that children devote to mastering learning tasks (e.g., “I am interested in the work I get to do in my classes”) (Shoshani et al., 2016b,c). Items are scored on a five-point scale, ranging from 1 (not at all) to 5 (yes, fits me well). This scale showed good internal consistency in the study (alpha coefficient = 0.81).

#### The Short Grit Scale (Grit-S; Duckworth and Quinn, 2009)

The short grit scale is a brief self-report version of the Grit Scale, which measures trait-level passion for long-term goals and perseverance. The scale consists of 8 items rated on a five-point scale from 1 (not like me at all) to 5 (very much like me), e.g., “I finish whatever I begin,” and “I often set a goal but later choose to pursue a different one.” The Grit-S has been reported to have good criterion validity for school children, and showed high longitudinal associations with GPAs (Duckworth and Quinn, 2009). The measure was translated to Hebrew for this study using forward and backward translation by experts in both languages. The scale yielded a good Cronbach’s alpha of 0.80 in this study.

### Data Analysis

A principal component analysis (PCA) was applied using SPSS 25.0 (SPSS Inc., Chicago, IL, United States) to examine the factor structure of the CSI-EC on the first sample. The criteria for identifying the CSI-C factors were based on Glorfeld’s version of parallel analysis (Glorfeld, 1995) in which the eigenvalues in the experimental data were compared to the 95th percentile of the distribution of random data eigenvalues, via O’Connor (2000) SPSS macro. A confirmatory factor analysis and hypothesis testing were conducted on the second sample data, using AMOS 25.0 (IBM Inc.). A model was considered to have acceptable fit if the following criteria were met: a CFI (comparative fit index) > 0.90; TLI (Tucker-Lewis index) > 0.90; RMSEA(root-mean-square error of approximation) < 0.08; and SRMR (standardized root mean square residual) < 0.08. In addition, CFI > 0.95; TLI > 0.95; RMSEA < 0.06; and SRMR < 0.05 are considered indicative of good fit (Brown, 2006; Hooper et al., 2008).

## Results

### Factor Structure of the CSI-C (Sample 1)

A PCA was conducted on the 120-item inventory to identify the factor structure of the CSI-C in the first sample. Parallel analysis was used to determine the number of character strengths factors to retain. The parallel analysis using the 95th percentile eigenvalues criterion indicated the extraction of 26 factors. Then, a PCA with a 26-factor solution was run with a direct oblimin rotation method, which allows the strengths factors to correlate. In this analysis, the best factor solution appeared to be a 24-factor solution, where two factors were composed of fewer than four items with a loading of 0.40. In addition, out of the 120 items, nine items were removed due to a high factor loading (above 0.60) for more than one factor; seven items were excluded due to lack of salience (factor loading < 0.4); the eight items that had the lowest factor loading of their respective strengths were dropped to obtain an equal number of four items on each scale and to shorten the questionnaire (see Appendix). Then the same extraction and rotation methods were performed on the remaining 96 items, which yielded 24 distinct character strengths factors that jointly accounted for 56.20% of the total variance (see Table 1). The correlations of each item with its respective factor ranged from 0.46 to 0.89. Table 1 also provides the alpha coefficients for the strengths subscales.

**Table 1:** Factor structure of the CSI-C (oblique rotation) (Sample 1).

Factor	Items	*α*	Eigenvalue	Variance (%)
1. Love	11,21,41,46	0.76	6.16	6.42
2. Social intelligence	1,3,5,48	0.75	4.94	5.15
3. Kindness	15,38,51,92	0.80	4.52	4.71
4. Zest	35,39,50,76	0.80	3.86	4.02
5. Appreciation of beauty	9,63,82,96	0.78	3.73	3.89
6. Creativity	8,10,16,42	0.74	2.91	3.03
7. Love of learning	70,74,83,87	0.88	2.70	2.81
8. Hope	18,27,44,57	0.73	2.50	2.60
9. Persistence	67,71,81,85	0.82	2.17	2.26
10. Leadership	31,36,77,88	0.83	2.08	2.17
11. Humor	14,37,54,80	0.83	1.82	1.90
12. Curiosity	34,78,84,90	0.90	1.78	1.85
13. Self-regulation	47,56,59,68	0.81	1.66	1.73
14. Perspective	13,17,58,93	0.85	1.39	1.45
15. Forgiveness	12,19,28,33	0.77	1.37	1.43
16. Open-mindedness	23,26,40,66	0.84	1.34	1.40
17. Prudence	6,29,53,73	0.86	1.26	1.31
18. Fairness	45,55,89,91	0.76	1.20	1.25
19. Gratitude	2,20,49,86	0.85	1.19	1.24
20. Teamwork	4,60,72,79	0.87	1.13	1.18
21. Bravery	22,43,52,62	0.88	1.10	1.15
22. Modesty	25,30,65,75	0.88	1.07	1.11
23. Authenticity	31,61,69,94	0.91	1.06	1.10
24. Spirituality	7,24,64,95	0.75	1.01	1.05

*N = 1,010; *α* = Cronbach’s alpha*.

A second-order factor analysis was conducted in the second stage, using the first-order 24 strengths scores as variables. Results of the parallel analysis indicated that four factors should be retained. A factor analysis with four factors specified which, when subjected to an oblimin rotation, resulted in a satisfying factor structure. The four factors explained 50.10% of the overall variance. A cutoff of a 0.40 factor loading was used to associate a strength subscale with a second-order factor.

The four factors that emerged from this analysis were highly similar to the factors originally extracted by Park and Peterson (2006b) in the VIA-Youth study, and were labeled Intellectual Strengths, Interpersonal Strengths, Temperance Strengths, and Transcendence Strengths (see Table 2). The means and standard deviations for the 24 strengths subscales appear in Table 2. The mean scores ranged from 3.29 to 4.43. Gratitude, teamwork, and kindness had the highest mean scores, whereas leadership, forgiveness, and love of learning had the lowest mean scores.

**Table 2:** Second-order factor structure and psychometric properties of the CSI-C (oblique rotation).

	*M*	*SD*	Interpersonal	Transcendence	Intellectual	Temperance
			strengths	strengths	strengths	strengths
Social intelligence	3.91	0.76	0.85	-0.10	-0.02	0.10
Teamwork	4.12	0.74	**0.78**	0.02	0.04	0.07
Leadership	3.29	1.11	**0.76**	0.13	-0.06	-0.22
Kindness	4.11	0.74	**0.74**	-0.04	0.08	0.08
Perspective	3.86	0.80	**0.74**	-0.04	0.17	-0.03
Love	4.02	0.80	**0.71**	0.12	-0.08	0.04
Bravery	3.99	0.80	**0.67**	0.10	0.04	0.00
Fairness	3.80	0.85	**0.61**	-0.06	0.09	0.28
Spirituality	3.65	1.08	-0.03	**0.77**	-0.01	-0.04
Gratitude	4.43	0.74	0.02	**0.75**	0.01	0.14
Hope	4.08	0.89	0.03	**0.74**	0.06	0.16
Zest	3.88	0.94	-0.01	**0.65**	0.21	0.11
Humor	3.86	1.07	0.20	**0.60**	0.08	-0.20
Curiosity	3.81	0.93	0.03	0.10	**0.79**	-0.05
Love of learning	3.55	0.98	0.02	0.03	**0.75**	0.13
Creativity	4.06	0.95	0.12	0.02	**0.74**	-0.11
Appreciation of beauty	3.97	0.92	0.00	0.09	**0.72**	0.02
Forgiveness	3.39	0.90	0.10	0.11	-0.21	**0.59**
Modesty	3.77	1.03	0.00	-0.02	0.20	**0.52**
Self-regulation	3.57	0.97	0.16	0.08	0.25	**0.50**
Authenticity	3.75	0.74	0.10	0.30	0.01	**0.46**
Prudence	3.96	0.87	0.26	0.05	0.27	**0.43**
Persistence	4.05	0.81	0.04	0.32	0.33	**0.41**
Open-mindedness	3.85	0.87	0.19	0.18	0.31	**0.40**
Eigenvalue			4.54	2.81	2.80	1.87
Percent of variance			18.92	11.73	11.67	7.79

*Factor loadings in bold are equal or greater than 0.4*.

### Confirmatory Factor Analysis (Sample 2)

**FIGURE 1:**
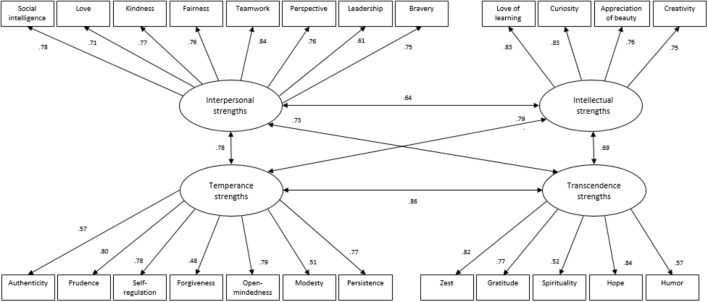
CFA model with standardized path coefficients (second sample).

A CFA was applied to examine the constructs emerging after the exploratory factor analysis on the 1,023 participants in the second sample, using the AMOS maximum likelihood estimation procedure. The CSI-C achieved acceptable fit based on the criteria specified by Hu and Bentler (1999), with the following mean fit indices: TLI = 0.903, CFI = 0.907, SRMR = 0.062, RMSEA = 0.053, confirming the 24 strengths model. A second CFA was performed to establish the higher order factor components. At the second-order level, the model subsumed the 24 first-order strengths factors under the four second-order factors (Intellectual, Interpersonal, Temperance, and Transcendence) (see Figure 1). The results indicated acceptable model fit indices with a TLI of 0.918, CFI of 0.933, SRMR of 0.055, and RMSEA of 0.062.

### Children’s Strengths and Social, Emotional, and Academic Outcomes

**FIGURE 2:**
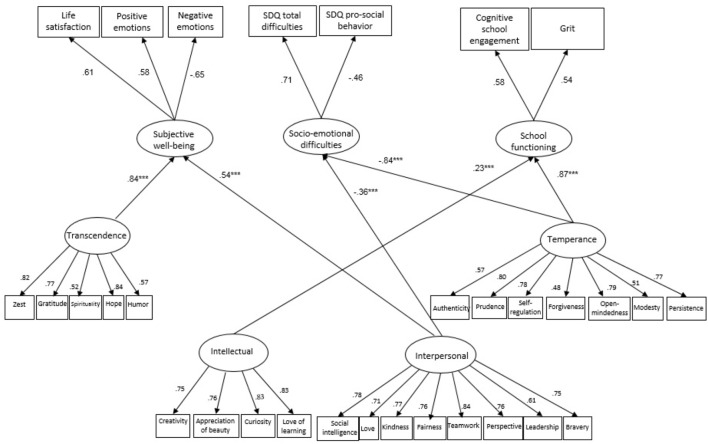
The association between children’s character strengths, SWB, socio-emotional difficulties and school functioning. SDQ, Strengths and Difficulties Questionnaire.

A structural equation model was constructed to test the study hypotheses on the associations of the character strengths factors with the mental health, subjective well-being, and school engagement indicators. The hierarchical model contained the 24 measured character strengths that loaded on the relevant second-order strengths factor related to the outcome variables. Three latent variables represented the predicted variables: subjective well-being, socio-emotional difficulties, and school functioning. Non-significant relationships between the latent strengths factors and the predicted variables were removed from this model, resulting in the model depicted in Figure 2. This model fit the data quite well (TLI = 0.90, CFI = 0.91, SRMR = 0.06, RMSEA = 0.05). Figure 2 depicts the parameter estimates for this model.

Significant negative relationships with socio-emotional difficulties were found for temperance strengths (β = -0.84, *p* < 0.001) and interpersonal strengths (β = -0.36, *p* < 0.001), whereas significant positive relationships with subjective well-being were found for transcendence (β = 0.84, *p* < 0.001) and interpersonal strengths (β = 0.54, *p* < 0.001). Finally, there were significant positive relationships between the intellectual (β = 0.23, *p* < 0.001) and temperance strengths (β = 0.87, *p* < 0.001) and children’s school functioning.

### Gender and Age-Related Differences

The gender and age comparisons appear in Table 3. *T*-test analyses revealed that girls reported higher average interpersonal (*t* = 4.13, *p* < 0.001, *Cohen’s d* = 0.27), transcendence (*t* = 2.89, *p* = 0.004, *Cohen’s d* = 0.18), intellectual (*t* = 5.09, *p* < 0.001, *Cohen’s d* = 0.31) and temperance (*t* = 5.08, *p* < 0.001, *Cohen’s d* = 0.33) strengths than boys. Girls also reported significantly higher levels of life satisfaction, prosocial behavior and cognitive engagement than boys (see Table 3).

**Table 3:** Means and standard deviations of the study variables by gender and age group (second sample).

	Gender							Age range						
	Total		Boys		Girls			7–8		9–10		11–12		
Sample size	1,023		506		517			340		348		335		
	*M*	*SD*	*M*	*SD*	*M*	*SD*	*Gender p*	*M*	*SD*	*M*	*SD*	*M*	*SD*	*Age p*
Interpersonal strengths	4.05	0.59	3.97	0.62	4.13	0.56	<0.001	3.96	0.59	4.13	0.56	4.18	0.62	<0.001
Transcendence strengths	4.03	0.71	3.96	0.76	4.09	0.66	0.004	3.96	0.67	4.16	0.62	4.14	0.76	0.001
Intellectual strengths	3.90	0.78	3.78	0.89	4.02	0.64	<0.001	3.88	0.80	4.02	0.71	3.98	0.76	0.09
Temperance strengths	4.04	0.65	3.93	0.70	4.14	0.58	<0.001	4.04	0.60	4.20	0.54	4.17	0.63	0.004
Life satisfaction	6.21	0.92	6.14	1.02	6.28	0.81	0.02	6.22	0.88	6.32	0.72	6.30	0.99	0.35
Positive emotions	19.95	4.00	19.78	4.11	20.12	3.89	0.17	19.66	4.23	20.04	3.83	20.82	3.89	0.003
Negative emotions	8.35	3.39	8.31	3.34	8.38	3.44	0.73	8.81	3.84	8.19	3.22	7.30	2.48	<0.001
SDQ total difficulties	8.49	5.54	8.24	5.48	8.76	5.59	0.14	11.28	5.15	9.36	4.55	8.02	5.36	<0.001
SDQ Pro-social behavior	7.46	3.09	6.96	3.29	7.97	2.79	<0.001	8.17	2.12	8.69	1.63	8.83	2.00	<0.001
Cognitive engagement	4.15	0.67	4.04	0.75	4.25	0.58	<0.001	4.04	0.70	4.24	0.57	4.20	0.70	0.002
Grit	3.49	0.65	3.47	0.70	3.51	0.59	0.33	3.58	0.69	3.49	0.52	3.41	0.59	0.005

In addition, ANOVA analyses showed significant differences in character strengths between the three age groups (7–8.9 years, 9–10.9 years, and 11–12.5 years), with significantly lower levels of interpersonal strengths, *F*(2,1020) = 10.06, *p* < 0.001, $\eta_{p}^{2}$ = 0.03, transcendence strengths, *F*(2,1020) = 6.82, *p* = 0.001, $\eta_{p}^{2}$ = 0.02, and temperance strengths, *F*(2,1020) = 5.63, *p* = 0.004, $\eta_{p}^{2}$ = 0.01, in the 7–8.9 age group compared to the older age groups. The younger age group (ages 7–8.9) was also lower than that of the older groups for positive emotions, pro-social behavior, and cognitive school engagement, and higher for negative emotions and grit (*p*s < 0.005).

## Discussion

This study provides preliminary validation of the Character Strengths Inventory for 7- to 12-year-old informants, which indicates that children can validly report on their own character strengths. Preliminary support for the validity and reliability of this children’s self-report measure is important in light of the inherent potential biases related to the use of parents as informants for children’s personality characteristics. Perhaps more importantly, the potential of utilizing children as informants about their own character using a relatively simple and straightforward survey methodology could accelerate investigations of the development of character during childhood. The findings also provide evidence for the expressions of all 24 character strengths of the VIA in middle childhood, and for the fundamental importance of childhood character strengths as important protective factors that can ameliorate socio-emotional difficulties and enhance the subjective well-being of elementary school children.

A factor analysis of the CSI-C revealed the presence of four reasonably coherent factors or underlying dimensions that constitute children’s character. These character dimensions can be viewed as conceptual categories through which young children acquire good character, and adults can promote their character development. One category represents the interpersonal aspect of children’s character, and includes strengths such as social intelligence, fairness, kindness, and love. These strengths are related to the child’s optimal interpersonal and group relationships (Park and Peterson, 2010). The second category of transcendence includes strengths that provide meaning to children’s lives, a positive interpretation of reality, and an emphasis on the “bright side” of life. It includes the strengths of zest, hope, spirituality, humor, and gratitude. The third category of intellectual strengths represents the cognitive qualities of the child that facilitate exploration, a passion for learning, creation of knowledge and innovation (Gillham et al., 2011) such as curiosity, love of learning and creativity. The fourth category of temperance is related to the child’s self-regulation of behaviors, and perseverance to achieve long-term goals (Berger et al., 2007). It includes strengths such as persistence, self-regulation, prudence, forgiveness and modesty. These four categories yield insights into the varied paths to good character during middle childhood, and highlight the need for a holistic approach that addresses cognitive, social, emotional, and transcendental features in character development efforts.

The character strengths factors in the CSI-C were associated with better mental health and subjective well-being, as expected given their conceptualization as positive and psychologically fulfilling (Peterson and Seligman, 2004). The temperance and interpersonal strengths made a significant contribution to the level of children’s mental health. Both of these have been described in the literature as resilience factors that promote mastery (e.g., adequate motivational and executive functioning, emotion regulation, and social support) in the face of environmental and social stressors (Slone and Shoshani, 2008a; Slone et al., 2009; Shoshani and Slone, 2016). It is also not surprising that the transcendence and interpersonal strengths were strongly associated with subjective well-being. These findings are consistent with the results of studies on adolescents using the VIA youth (Park, 2004; Gillham et al., 2011; Shoshani and Slone, 2013; Weber et al., 2013), and suggest that character strengths have considerable importance for positive development of children not only as buffers against mental health difficulties but also as enabling resources that promote children’s thriving and flourishing. A relatively new finding in this area of research is the contribution of children’s intellectual and temperance character strengths to their cognitive engagement and grit. Grit is typically operationalized by two facets: consistency of interest and perseverance of effort (Duckworth and Quinn, 2009). Our findings demonstrate that both the passion for learning and self- regulative processes are an essential part of children’s character that provide a venue for motivated and sustained engagement in learning. As a whole, these findings imply that good character in childhood is comprised of a balanced set of social, emotional, and cognitive qualities that allow children to flourish, transcend misfortunes, and achieve a good and meaningful life.

Analyses also revealed several significant gender differences. For all the strengths factors, the girls reported higher ratings than the boys. Several studies have examined and reported gender differences in character strengths (Biswas-Diener, 2006; Shimai et al., 2006; Linley et al., 2007; Miljković and Rijavec, 2008). However, it seems difficult to find a consistent pattern in these differences except for higher ratings on love and kindness for adult females (Shimai et al., 2006; Linley et al., 2007; Miljković and Rijavec, 2008). These authors accounted for these patterns in terms of differences in particular gender roles and expectations in specific cultures. These findings accentuate the need for comparative and cross-cultural research on children’s character strengths.

Several age differences were also observed. Noticeable differences were found between 7- and 8.9-year olds as compared to 9- to 12-year olds, including lower levels of interpersonal, transcendence, temperance strengths, emotional well-being and school engagement. Because cognitive and emotional maturity increase with age, children in late childhood (9–12) as compared to the earlier childhood years may more easily establish relationships, regulate their emotions and behaviors, and may be more active in constructing meaning and positively interpreting their experiences (Shoshani and Russo-Netzer, 2017). However, the relatively small number of studies that have dealt with character strengths and subjective well-being has made it difficult to interpret these age trends, and indicate that much more research is needed to better understand character development in various cultures and its relationship to children’s subjective well-mental health.

This study has several limitations that deserve consideration. Methodologically, this study relied on single-source data from children’s self-report questionnaires; therefore, common method variance may have inflated the observed correlations between the study measures. In future research it would be desirable to rely on multi-informants (parents, teachers, and children), and to use a variety of research methods and means of data collection (laboratory-based behavioral tasks, observations, and questionnaires) to achieve a more comprehensive picture of children’s character strengths. In addition, due to the cross-sectional nature of this study design, causal inferences about the relationship between children’s character strengths and their mental health and well-being cannot be made. Longitudinal studies are required to describe changes over time in children’s character and mental health factors, and to investigate the evolution of character from childhood to adolescence.

Finally, this study was conducted in an Israeli context, in a setting of protracted conflict and war that may affect children’s well-being (Shoshani and Slone, 2008; Slone and Shoshani, 2008b, Slone and Shoshani, 2014a,b). Further comparative research should be carried out in different countries to investigate underlying cultural and contextual biases. Although the VIA authors argued that these character strengths are ubiquitous and perhaps universal values (Park et al., 2006), there is a clear need to examine this argument with cross-national data. Further efforts are needed to better understand the similarities and differences in the ways in which these strengths are manifested in children and the correlates and consequences of these 24 strengths in different nations and cultures.

In addition, as mentioned previously, the inconsistencies of previous studies on the factorial structure of the VIA inventories have raised questions about the cultural invariance of the six-virtue cluster model proposed by Peterson and Seligman (2004) (e.g., Duan et al., 2012; Wong, 2013). Duan et al. (2012) proposed a two stage solution to this problem. They suggested an exploration of the virtue factor based on the 24 strengths across different cultures with comparable methods and samples as a first step, and a development of a stand-alone classification of the virtues obtained in each culture with culturally adapted instruments to measure them, as a second step. In conclusion, further studies still need to be done, to compare the factor structure and the psychometric properties of the CSI-C across different cultures.

Despite these limitations, the findings have vital importance for the fields of character strengths. This study provides a valuable and comprehensive tool for practitioners and researchers examine character strengths in children in broad fields of interest such as positive psychology, character education, and social-emotional learning. On a psychological-clinical level, the use of a character measurement instrument can be an important addition to the classic diagnostic assessment battery designed to identify symptoms, by focusing on children’s strengths and protective factors. More importantly, it can emphasize the positive side of children’s experiences, alongside understanding and ameliorating psychological symptoms and distress. In a more general way, this study also provides a framework for an assessment of good character in children. A sensitive awareness of the main dimensions of character in education and family settings during the period of personality formation in childhood is of critical importance to the development of healthy, resilient, capable, and happy children.

## Ethics Statement

This study was carried out in accordance with the recommendations of the Herzliya Interdisciplinary Center Academic Ethics Committee and the Israeli Ministry of Education, and obtained informed consent from all participants. Parents gave written informed consent in accordance with the Declaration of Helsinki. The protocol was approved by the Israeli Ministry of Education’s Ethics Committee and the Herzliya Interdisciplinary Center Academic Ethics Committee.

## Author Contributions

All authors listed have made a substantial, direct and intellectual contribution to the work, and approved it for publication.

## Conflict of Interest Statement

The authors declare that the research was conducted in the absence of any commercial or financial relationships that could be construed as a potential conflict of interest.

## Appendix

**Table A1:** The Character Strengths Inventory for Children; CSI-C.

Character strengths	Items	
Social intelligence	1.	I play well with other children and almost never fight.
	48.	I usually know how to prevent problems and fights with others.
	5.	I usually know how to say the right thing that will make others feel good.
	3.	I know how to solve problems in a way that makes everyone feel okay.
Love	11.	I show a lot of warmth and love toward my friends and family.
	21.	There is someone who will listen to me when I have a problem.
	41.	I am not afraid to tell my relatives and friends that I love them.
	46.	I feel loved.
Kindness	51.	When I see another child in distress or encountering a problem, I try to help.
	15.	People think I am considerate of my surroundings, nice to others, and kind.
	92.	I always volunteer to help when I see someone in need.
	38.	I do nice things for others on my own initiative, without being asked to do so.
Fairness	45.	I relate to all children fairly, even if I do not like them.
	89.	When candies or treats are handed out in school or at home, I make sure that everyone gets an equal share even if I do not like everyone equally.
	55.	I relate to all children fairly, even if they are not nice to me.
	91.	I don’t like to discriminate so I make an effort to treat everyone equally.
Teamwork	72.	I consider the desires and needs of other children when playing in a group.
	60.	I am very loyal to my group and/or my friends.
	4.	In situations when I am placed in a group, I get along well with the other children in my group.
	79.	I know how to include other children and work together on tasks when I am in a group with other children.
Perspective	17.	I know how to make decisions in a wise, level-headed manner.
	93.	My friends consult me before they make an important decision.
	58.	People say I am mature compared to other children my age.
	13.	In situations in which things do not go the way I want, I am able to make wise decisions.
Leadership	88.	I tend to be the leader in games or athletic activities with other children.
	31.	I tend to be the leader of the group and the other children follow me.
	77.	Other children see me as a class leader, listen to me, and trust me.
	36.	I’m perceived as a leader when I am around other children.
Bravery	43.	I do not hesitate to express my opinion or behave differently from my friends when I think this is the right thing to do.
	52.	I do the right thing even if others might make fun of me.
	22.	Even when I am scared to do something, I will do it if I know it is the right or worthwhile thing to do.
	62.	When another child is being hurt unjustly, I will rush to that child’s defense.
Love of learning	87.	I am enthusiastic and excited when I learn something new.
	83.	I enjoy places and situations where I am introduced to new information, such as movies about science and nature or visiting a museum.
	74.	I love learning new skills.
	70.	I take advantage of opportunities to learn something new.
Curiosity	90.	When there is a conversation about a topic I am unfamiliar with, I immediately want to know more about it.
	84.	I am always interested in discovering new things that I didn’t know before.
	34.	I love to explore the world around me and discover new things.
	78.	I am interested and full of questions about things I am unfamiliar with.
Appreciation of beauty	96.	I like to stop and to look at beautiful things around me, such as flowers, butterflies, and beautiful landscapes.
	63.	I enjoy good music or beautiful works of art.
	42.	I feel happier when I see a beautiful work of art or listen to nice music.
	82.	I like beautiful things.
Creativity	16.	I have a lot of ideas and I am very creative.
	10.	I love inventing and creating new things.
	8.	I have many creative ideas.
	9.	I am full of new ideas about things to do or make.
Prudence	29.	I know how to stop or avoid situations that endanger me.
	73.	I am careful not to do something I will regret later.
	6.	I distance myself from situations and children that are liable to get me into trouble.
	53.	When I make a decision I consider the advantages and disadvantages of both sides.
Self-regulation	47.	I am very calm and I generally do not have tantrums or lose control.
	68.	I have a lot of patience.
	59.	I am capable of waiting if asked to do so, even when I very much want to do something at the time.
	56.	I am able to control my anger in an effective way.
Forgiveness	12.	If a child hurts my feelings it is difficult for me to continue playing with him.
	19.	It is difficult for me to forgive children who have hurt me in the past.
	50.	I often stay angry with other children even after they apologize.
	28.	I usually stay angry with other people even after they apologize.
Open-mindedness	26.	I am open and attentive to opinions other than my own and can be swayed by them.
	40.	Even when I do not want to do something, when its importance is explained to me, I am usually persuaded to do it.
	66.	I listen to the opinions and advice of others before deciding what to do.
	23.	I respect my friends’ opinions and thoughts, even when I don’t agree with them.
Modesty	75.	I do not brag about my achievements when I am at school or at home.
	25.	I am not a showoff.
	30.	I am not condescending and I don’t think I am better than my friends.
	65.	I do not typically tell other children that I am better than they are.
Persistence	81.	If I take on a responsibility, I do everything I can to fulfill it.
	71.	I am able to sit for a long time to complete a project I decided to do, such as an art project, building with Legos, or a complex puzzle.
	67.	Even in situations that are difficult for me, I do not give up or stop in the middle of things that are important to me.
	85.	I know how to work very hard and to invest a lot of effort into things that are important to me, such as hobbies and clubs.
Zest	76.	I am a happy person and get excited about things easily.
	35.	I think life is exciting.
	39.	I know how to enjoy and be enthusiastic about the small things in life.
	33.	I am full of joy of life and vivaciousness.
Gratitude	20.	I mostly feel really lucky for what I have in my life.
	86.	I know how to appreciate and say thank you for food prepared for me or a gift that I receive.
	2.	I know how to appreciate the good things that happen in my life.
	49.	I express gratitude for the good things that are done for me.
Spirituality	95.	When things that are not good happen to me, my religious beliefs help me feel better.
	24.	I believe in God or a supreme power that protects and directs me to do the right thing.
	7.	I love and am drawn to spiritual things such as praying, doing techniques to develop the imagination, or breathing and relaxation techniques.
	64.	I feel better when I pray.
Hope	44.	When I am in new situations, I generally assume that good things will happen to me.
	27.	Even when things are hard for me, I believe that ultimately things will be good.
	57.	Even when bad things happen to me, I remain full of hope.
	18.	When I do not succeed at something, I believe I will do better the next time.
Humor	54.	I am good at making other children and adults laugh.
	37.	My jokes and the fact that I am funny helps me cope with social situations.
	14.	People tell me I am funny.
	80.	Making other children laugh is something I do well.
Authenticity	94.	I do not usually lie.
	32.	I am generally truthful and I should be believed in most cases.
	61.	I tell the truth even when this means I will not get what I want.
	69.	I tell the truth even when I do things that are not OK.
